# Kondo effect and enhanced magnetic properties in gadolinium functionalized carbon nanotube supramolecular complex

**DOI:** 10.1038/s41598-018-26428-y

**Published:** 2018-05-23

**Authors:** S. Ncube, C. Coleman, A. Strydom, E. Flahaut, A. de Sousa, S. Bhattacharyya

**Affiliations:** 1 Nano-Scale Transport Physics Laboratory, School of Physics, and DST/NRF Centre of Excellence in Strong materials, University of the Witwatersrand, Johannesburg, South Africa; 2 Highly Correlated Matter Research Group, Department of Physics, University of Johannesburg, Auckland Park, 2006 South Africa; 3 Max Planck Institute for Chemical Physics of Solids, Nöthnitzerstr. 40, D-01187 Dresden, Germany; 4 CIRIMAT, Université de Toulouse, CNRS, INPT, UPS, UMR CNRS-UPS-INP No. 5085, Université Toulouse Paul Sabatier, Bât. CIRIMAT, 118, route de Narbonne, 31062, Toulouse, cedex 9 France; 5 School of Chemistry, University of the Witwatersrand, Johannesburg, South Africa

## Abstract

We report on the enhancement of magnetic properties of multiwalled carbon nanotubes (MWNTs) functionalized with a gadolinium based supramolecular complex. By employing a newly developed synthesis technique we find that the functionalization method of the nanocomposite enhances the strength of magnetic interaction leading to a large effective moment of 15.79 *µ*_B_ and non-superparamagnetic behaviour unlike what has been previously reported. Saturating resistance at low temperatures is fitted with the numerical renormalization group formula verifying the Kondo effect for magnetic impurities on a metallic electron system. Magnetoresistance shows devices fabricated from aligned gadolinium functionalized MWNTs (Gd-Fctn-MWNTs) exhibit spin-valve switching behaviour of up to 8%. This study highlights the possibility of enhancing magnetic interactions in carbon systems through chemical modification, moreover we demonstrate the rich physics that might be useful for developing spin based quantum computing elements based on one-dimensional (1D) channels.

## Introduction

Over many years there have been various attempts to investigate the interaction between magnetic metal clusters and conductors to develop spintronic devices based on ferromagnetic metal and carbon nanotube complexes^1^. Carbon nanotubes (CNTs) are ballistic conductors which exhibit exciting quantum transport phenomena such as Luttinger liquid behaviour^2–4^ and the Coulomb Blockade^5–7^. Yet this material suffers from weak spin orbit interaction which limits the observation of strongly correlated resonant transport features including the Kondo effect^8–10^, Andreev reflection^11,12^, and the Majorana zero modes^13^ which have been reported in a range of other nanowires. Kondo resonance studies were pioneered in CNT and other low dimensional systems through the observation of the zero-bias anomaly peak^8–10,14^ which remains elusive in a CNT network. Much work has been done to find a material that serves as a good electron conductor as allowing for spin mediation. It is well known that chirality of an individual CNT determines the successful spin transport which limits the spintronic application of this material. However, bundles of CNTs working as a multichannel system can overcome this problem if CNTs can be doped with a rare earth element and a link between the metal islands through the carbon backbone is established. Here we show a new synthesis route of networking CNTs through the clusters of a gadolinium (Gd) based complex that effectively forms a multichannel system that results in exciting electronic transport features related to Kondo effect and effective enhancement of spin-orbit coupling. Previous attempts in CNT based devices show that both localization and tunnelling effects^15^ can have dominant features in the transport. Although single walled carbon nanotube (SWNTs) networks and individual tubes can exhibit either semiconducting or metallic behavior^15^, bundles and multiwalled carbon nanotube generally show an activated transport mechanism where fluctuation-assisted tunnelling effects dominate the transport^15^. These cotunnelling phenomena have also been shown to lead to spin accumulation^16^ and can drastically enhance tunneling magnetoresistance (TMR)^17^ and lead to enhanced spintronic device properties.

It is well known that due to weak spin orbit coupling the spin relaxation time of carbon systems, particularly CNTs, is relatively large (approximately 1 µs)^18^. SWNTs and MWNTs are not intrinsically magnetic but do show diamagnetic susceptibility that increases linearly with diameter^19,20^ when a magnetic field is applied. Interestingly, it has been demonstrated that the susceptibility of MWNT is highly anisotropic with regard to the orientation of the applied field and that the susceptibility is less diamagnetic with fields parallel to the CNT axis than in the perpendicular orientation^21,22^. Due to these favourable properties there have been many studies on the use of CNTs for spin valve devices, these typically involve the coupling of a CNT to ferromagnetic leads and injecting spin polarized current through the CNT and measuring the response^23–28^. There have also been investigations on supramolecular spin-valve devices based on individual SWNT non-covalently functionalized with molecular magnets along the surface of the CNT^29^. The advantage of using such supramolecular devices is that the specific magnetic molecules attached to the CNT can be tailored to exhibit the desired magnetic properties^30,31^. The non-covalent functionalization preserves the integrity of the structure of the CNT but spin interaction between localized magnetic moment and conduction electrons is weakened. There are however a range of possible routes for the attachment of molecules to CNTs, ranging from weakly attached grafting to strongly attached covalent bonding of the molecule onto the CNT^32^. A comparative study of the effect of the method of nanomagnet attachment on the magnetic response is yet to be made. Filling of CNTs is an alternative route to modifying their properties. There have been many reports on the filling of CNTs with a range of materials such as metals (Fe, Co, W), chalcogenides (Te, Se) and even other carbon structures such as buckyballs^33–35^.

In this work the properties of a supramolecular complex synthesized using a chemical method of incorporating gadolinium magnetic nanoparticles into a MWNT system via a diethylene triamine pentaacetic acid (DTPA) molecular complex that has been widely studied as a magnetic resonance imaging (MRI) contrast agent^33^ are investigated. The focus is on the covalent attachment of a Gd-DTPA complex to the outer wall of the MWNTs. Gd^3+^ is of particular interest due to its high magnetic moment = 7.94 *μ*_B_ which is expected to allow for spin correlations in the MWNTs^36^. The attachment of Gd^3+^ to CNTs has been explored before, yielding interesting features such as the observation of superparamagnetism as well as first order paramagnetic-ferromagnetic transitions^37^. In this work it is shown that the functionalized MWNTs exhibit a finite magnetic coercivity and remanence at room temperature. Structural characterization is used to establish the origins for the difference in magnetic behavior from previous reports. The unexpected properties observed for the functionalized sample prompted electronic transport studies on devices fabricated from a network of the functionalized MWNTs. This was done to determine the effect of the magnetic Gd-DTPA complex on the quantum transport of the MWNTs which is useful for high speed electronics and is an extension of an earlier study^38^. A saturating resistance was found as the temperature is lowered below 10 K. These features are attributed to the Kondo effect in a spin electron correlated carbon system where spin flipping events can lead to spin switching of the (tunnel) magnetoresistance.

## Results

### Structural characterization

**Figure 1:**
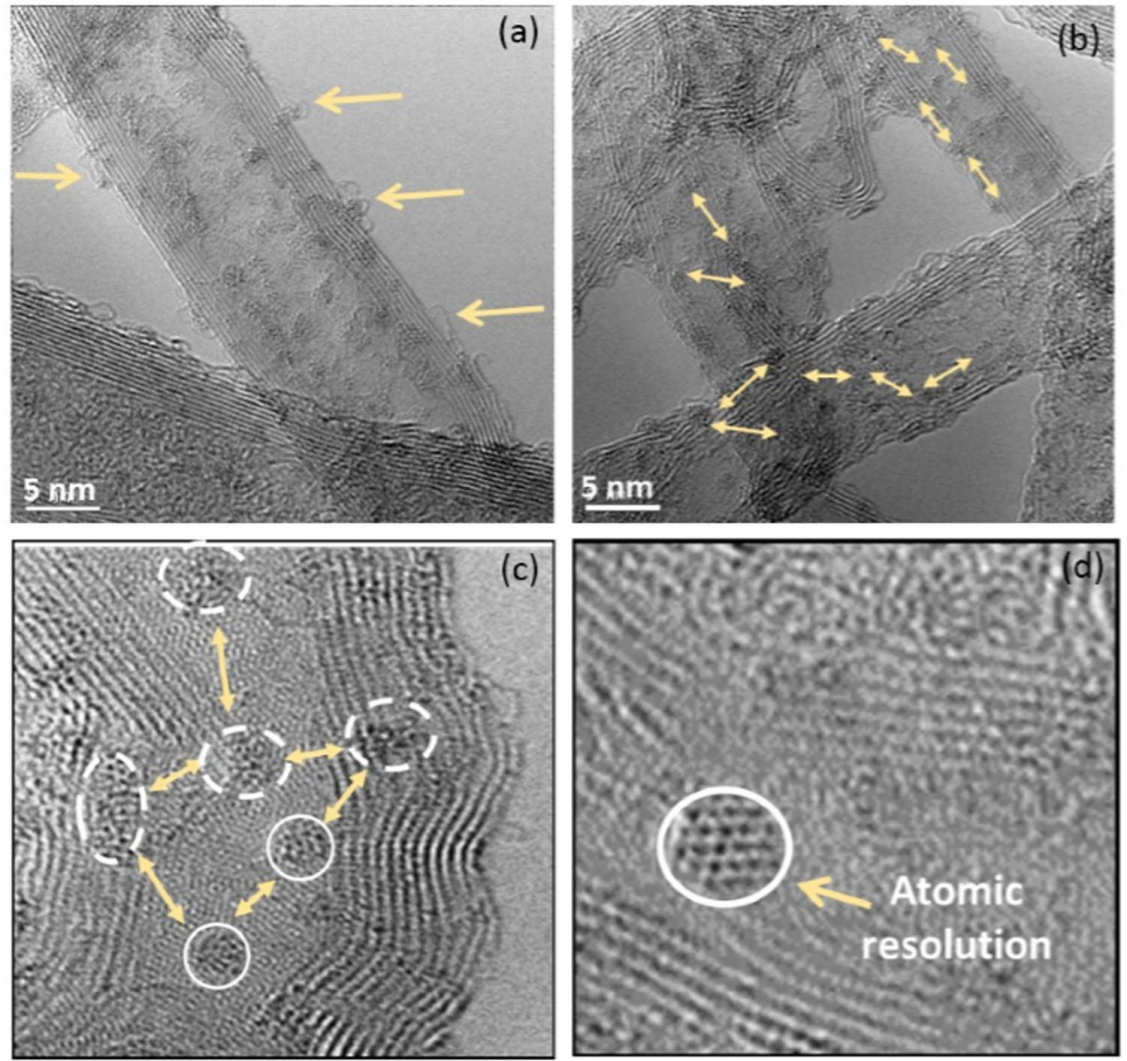
HRTEM image of the Gd-DTPA functionalized MWNTs. (**a**) The red arrows indicate regions along the nanotube that have strand like filament structures, believed to be a result of the DTPA ligand attachment. These strands are generally in close proximity to the multiple Gd^3^+ centers. (**b**) The MWNTs show a fairly homogenous distribution of the Gd-DTPA attachment throughout the sample, forming a network-like cover of the outer wall; distances between centres are indicated by the yellow arrows. (**c**) Multiple Gd-DTPA centres separated by a distance greater than 10 nm are indicated by the arrows. (**d**) Morphology of aggregated centres viewed under higher magnification circled.

**Figure 2:**
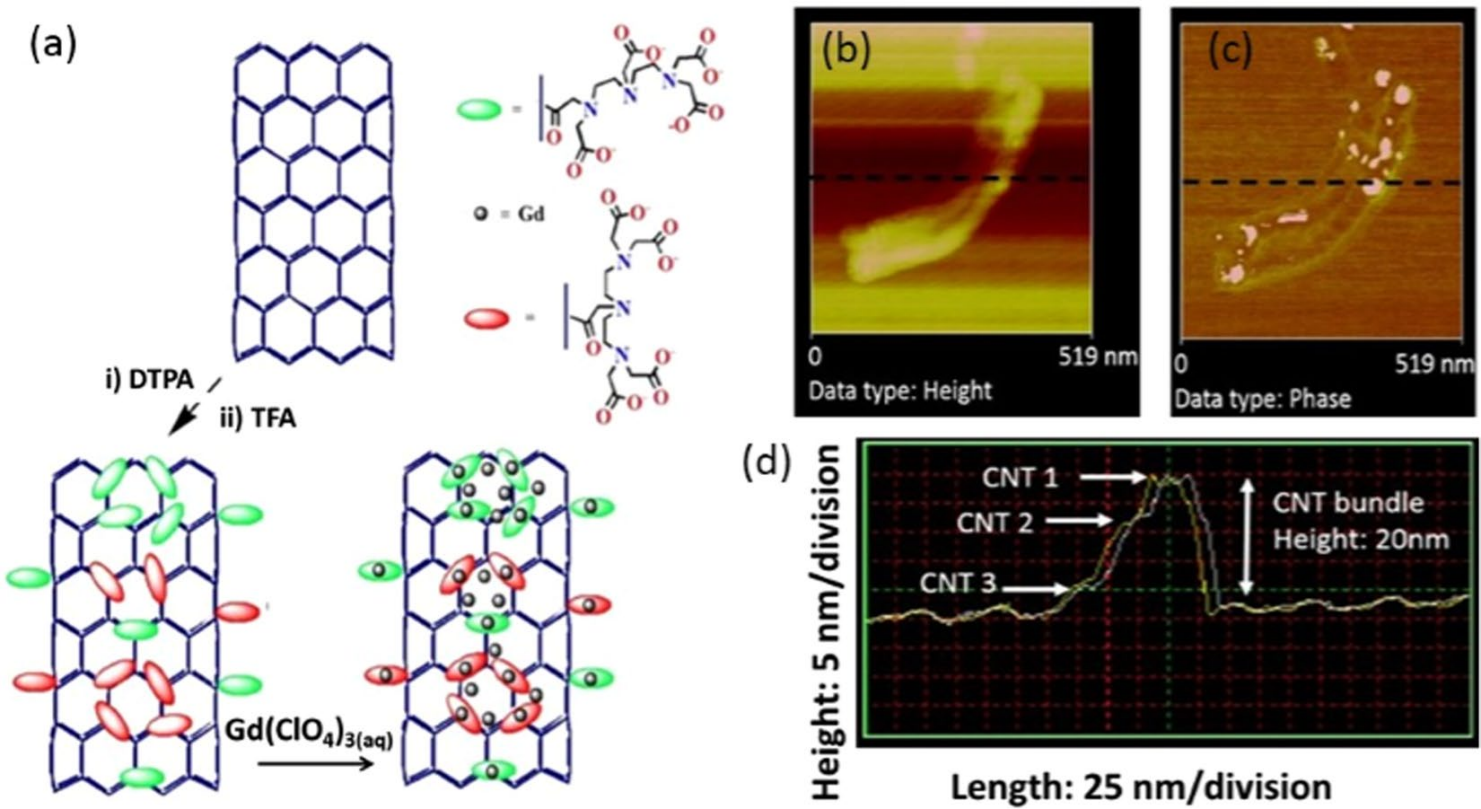
Investigation of the existence of magnetic domains on the CNTs. (**a**) Schematic illustration of MWNT functionalization with subsequent DTPA chelation of paramagnetic ion (Gd^3+^); varied attachment of the Gd-DTPA complex occurs through brachial carboxylate functionalities of the DTPA molecule (**b**) AFM in non-contact mode showing a small bundle of MWNTs nanocomposite. (**c**) The MFM scan over the same area as in ‘b’ showing small bright features along the surface of the MWNT lengths, which indicates magnetic interaction with the probe tip due to existence of magnetic domains. (**d**) The height profile of a line scan (dotted line in ‘b’ and ‘c’ across the MWNT bundle. Bundle consists of at least three nanotubes of diameters estimated to be 10 nm with a bundle height profile of approximately 20 nm.

The functionalized MWNTs (Fig. 1a–d) shows that Gd^3+^ centres are accommodated by fibril and spherical shaped nanostructures of approximately 2 nm in diameter, with a relative uniform distribution in close proximity but not continuous coverage of the outermost surface of the MWNTs. Atomic resolution of the Gd-DTPA aggregate can be seen on the high resolution transmission electron microscopy (HRTEM) image (Fig. 1d). It was found that the Gd-Fctn-MWNTs contain less than 0.2 mass percent cobalt (catalyst. material), with 14.57% of the mass determined to be gadolinium. A strategic approach towards covalently grafting a molecular paramagnetic species to MWNTs involves the use of DTPA molecules as suitable chelators covalently linked to the MWNT wall (see Fig. 2a). Synthetic methodologies have exploited DTPA dianhydride as a starting reagent or have resorted to alternative bridging chains for covalent binding of the DTPA chelate to nanotubes. The former compromises chelation of the paramagnetic metal ion (Gd^3+^) and inevitably is accompanied by a decrease in the ligand coordination number of the paramagnetic coordination polyhedron.

In such instances, the coordination polyhedron is completed by additional aqua ligands. The latter incorporates the chelate with increased flexibility affords higher rotational degrees of freedom to the Gd-O vector; affecting the possible transfer of magnetic information. An alternative approach considers a more suitably rigid grafting of the Gd-complex to the CNT. In this work acylation of the nanotube surface using a polyaminocarboxylate chelate bearing several carboxylate functionalities is investigated. Grafting of DTPA chelates in this manner affords two possible binding modes in which the octadentate nature of the DTPA chelate is potentially retained. Octadenticity is closely associated with lower order (ML) Gd^3+^ complexes, while higher order (M_x_L_y_)^n+^ complexes of decreased ligand denticity, enables completion of the coordination polyhedron by increased hydration or through proximal complex aggregation (Fig. 2a). AFM image in the non-contact mode shows a typical bundle of functionalized MWNT (Fig. 2b), in MFM imaging mode features resulting from the magnetic interaction of sample and probe tip are clearly observed on the surface of the MWNT corresponding to magnetic domains due to the presence of the Gd-DTPA (Fig. 2c). A line profile of the atomic force microscopy (AFM) and magnetic force microscopy MFM scans corresponding height profiling from the image shows peaks denoting a bundle of three CNTs (Fig. 2d) which collectively form a bundle of 20 nm high.

### Raman and FTIR spectroscopy

**Figure 3:**
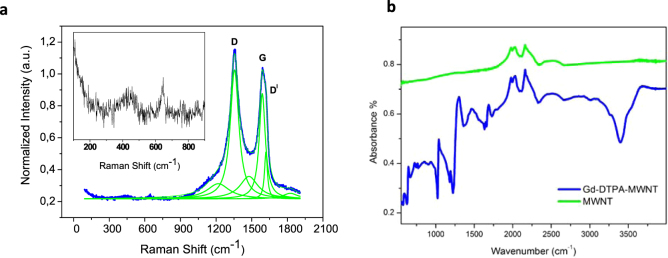
Raman and FTIR analysis. (**a**) Raman spectrum for the functionalized composite material showing the deconvolution of the G and D peaks. The inset is an indication of the low wavenumber vibrational modes attributed to the presence of Gd. (**b**) FTIR spectra for the Gd-DTPA complex and the multi-walled nanotubes.

As shown in Fig. 3a the functionalized MWNTs exhibit well pronounced G and D-bands as expected for MWNTs^39^ with some disorder due to the chemical treatment. The functionalized sample shows a large D-peak (integrated intensity: *I*_*D*_) compared to the G-peak (integrated intensity: *I*_*G*_), (*I*_*D*_/*I*_*G*_ = 1.26), this is an indication of higher levels of disorder. The graphitic crystallite size between Raman active defects (*L*_*a*_) in the samples is calculated using the Tunistra Koenig relation^39^ where *C*(*λ*) is a constant that depends on the excitation wavelength (*λ*). For *λ* = 514 nm, *C*(*λ*) ~ 4.4 nm.

1 $$\frac{{I}_{D}}{{I}_{G}}=\frac{C(\lambda )}{{L}_{a}}$$

We find that the functionalized MWNTs have a crystallite size of 3.57 nm, in good agreement with Gd-DTPA functional group distribution observed in HRTEM. An upward shift of the Raman G-peak position is observed compared to the pristine MWNTs (1582 cm^−1^). The shift in G-peak position of the Gd-functionalized sample is (Δω_G_ = 7 cm^−1^) and upon deconvolution it was determined that the asymmetry in the line width was due to the so called D′ peak situated at 1620 cm^−1^. Like the D-peak, the D′ peak is an indication of disorder and is commonly observed in functionalized MWNTs. The width of the G and D-peaks shows a broadening compared to the pristine case, an increase in G peak full width half maximum (G-FWHM) is an indication of increased disorder^39^. As expected, the functionalized sample showing a high D-peak intensity also exhibits a high broadening of the G- and D-peaks. The deconvolution of the G and D-peaks of the functionalized sample also identified two broad low intensity bands situated at 1218 and 1476 cm^−1^. The two features have been observed before in disordered graphite samples. The functionalized MWNT sample with the Gd-DTPA bonded to the outer tube wall also show multiple peaks of small intensity between 200 and 1000 cm^−1^. These peaks are expected to be a signature of the Gd complex attached to the CNTs as they are not typical features of MWNT Raman spectrum. Comparing the infrared spectrum of the MWNT to that of the Gd-Fctn-MWNT affords evidence for functionalization of the pristine MWNT surface (Fig. 3b). The spectrum of Gd-Fctn MWNT displays peaks in the range 3008–3550 cm^−1^ arising from O-H and C-H vibrations; the former may include vibrations of water molecules residing in the MWNT. Stretching vibrations of the carboxylate functionalities are evident from peaks in the range 1100–1740 cm^−1^. In particular the peaks at 1636 cm^−1^, 1661 cm^−1^ and 1734 cm^−1^ confirm the existence of three different carbonyl environments. The FTIR vibrational modes observed in the Gd-Fctn-MWNT are presented in the supplementary information Table S1. Elemental analysis of the CNT nanocomposites was carried out using Energy Dispersive Spectroscopy (EDS) shown in supplementary information Figure S1. It was confirmed that Gd^3+^ was present in the samples through the observation of the prominent peaks observed at 1.1, 6.05 and 8 keV; these are attributed to M, Lα_1_ and Lβ_1_ excitations specific to gadolinium.

### Magnetization characterization

**Figure 4:**
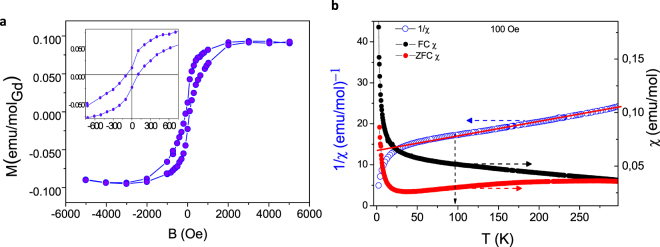
Magnetization studies. (**a**) Hysteretic behaviour of Gd-Fctn-MWNT sample showing weakly ferromagnetic behavior. Inset: Magnified region demonstrating magnetic remanence and coercive field. (**b**) FC and ZFC susceptibility and inverse susceptibility plots for Gd-Ftcn-MWNT composite at 100 Oe.

Figure 4a shows the magnetic moment as a function of the applied field of Gd-Fctn-MWNTs (see also supplementary information Figure S2). The nanocomposite exhibits a definite magnetic hysteretic behavior between forward and reverse field sweeps with a coercive field of 185 Oe suggesting possible single domain behaviour of the Gd^3+^ nanoparticle and a magnetic remanence of approximately 0.013 emu/mol_Gd_. The functionalized composite clearly demonstrates hysteresis closely related to a ferromagnet most likely due to the presence of the rare earth element. To further probe the nature of the magnetic behavior a study of the magnetic susceptibility was conducted between 300–1.7 K. Magnetization under field cooled (FC) and zero-field cooled (ZFC) procedures shows a difference in terms of curvature with decreasing temperature however the trend is qualitatively the same (Fig. 4b). As mentioned in the introduction there are several reports on Gd incorporated carbon nanotubes, either through filling^40^ or through chemical functionalization^41,42^, which exhibit super paramagnetism. This is clearly not the case in this system as no blocking temperature can be identified in the susceptibility of the Gd-Fctn-MWNT composite shown in Fig. 4b. The inverse susceptibility of the FC data set was plotted as a function of temperature to determine the coupling mechanism. The composite shows linearity down to 100 K after which the susceptibility increases (and inverse susceptibility decreases). The functionalized MWNTs have a Weiss temperature of −413 K. In general, the antiferromagnetic exchange requires the existence of interaction between spin sublattices of different spin orientation which in this system are likely due to the DTPA complex and itinerate electrons of the MWNTs which mediate the antiferromagnetism via the Ruderman–Kittel–Kasuya–Yosida (RKKY) interaction. RKKY has been reported in other magnetized carbon systems and it is a well-established fundamental interaction in spin polarized environments^43,44^. The inverse susceptibility plot was also used to determine the Kondo temperature T_K_ for this nanocomposite. It is extrapolated from the point where the inverse susceptibility plot starts to deviate from linearity^45^, estimated to be 98 K as shown in Fig. 4b. When calculating the effective moment in terms of the molar concentration of the Gd^3+^, which is determined from the elemental analysis, an enhanced effective moment of 15.79 µ_B_ was established. This value of the calculated effective moment is much larger than the effective moment of the Gd-DTPA complex (8.7 µ_B_ see supplementary information). The large value reported here is a clear indication of interactions between the Gd-DTPA chelates along the MWNTs surface, this is likely a result from the close proximity of the [Gd-DTPA]^n+^ entities which allows for complex aggregation with enhanced effective moment^40–42^. This aggregation is evident in the HRTEM as indicated in Fig. 1a–d. Not surprisingly the effective moment calculated here is similar to that reported for supramolecular fullerenes with endohedral trimetallic nitride clusters (Gd_3_N@C_80_), which was found to be 23 *µ*_B_ and it was shown that Gd_3_N clusters allows for ferromagnetic coupling and a largely enhanced moment^46^. It is believed a similar scenario is at play in the present study. These findings clearly demonstrate how the magnetic properties of composite can be modified by controlling the chemical functionalization process and that a mesoscopic magnetic correlated state can be observed.

## Electronic Transport

**Figure 5:**
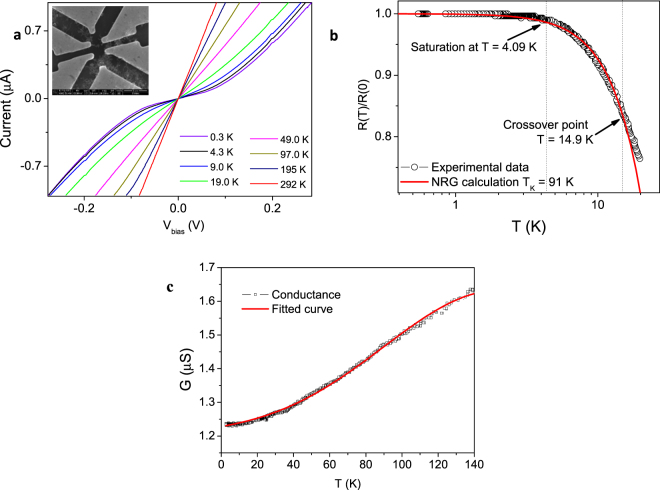
Electronic transport properties. (**a**) *I-V* sweeps from a temperature range of 300 mK up to 300 K. The inset and SEM image of the device used. (**b**) Temperature dependent normalized resistance showing a steady increase as temperature decreases and eventual saturation from 4 K, the red line is a fit to equation 3. (**c**) Conductance as function of temperature up to 300 K, the redline there is a fit to equation 2.

Figure 5a shows the *I-V* characteristics of the Gd-Fctn-MWNT network device at various temperatures ranging from 300 mK to room temperature and the inset shows a typical device used in this work where the Gd-Fctn-MWNT bundles aligned between the electrodes. *I*-*V* characteristics change progressively over the temperature range and a large deviation from linearity is clearly seen at 300 mK. The strong nonlinearity at low temperatures is an indication of the opening of a band gap, possibly due to the Coulomb blockade or charging effects. Figure 5b shows the variation of normalized resistance with temperature. The conductance was measured as a function of temperature for the same range (as shown in Fig. 5c and shows a steady decrease to approximately 4 K and then saturates below this temperature. Analysis of the temperature dependent resistance indicates that the Gd-Fctn-MWNT networks do not follow variable range hopping^15^ which is the expected mechanism for carbon nanotube devices of this type. This was concluded after failure to linearize the logarithmic normalized conductance as a function of *T* ^β^, where *β* is a critical exponent representing the dimension scale of the hopping (see supplementary information Figure S3). The devices do however display similar trends to those reported for thicker SWNT networks^15^ and conducting polymers^47,48^ that suggests interrupted metallic conduction mediated by fluctuation induced tunnelling (FIT). A nonlinear fit to the data set gives a relation similar to that presented in ref.^15^.

2 $$\sigma (T)={\sigma }_{1}T+{\sigma }_{2}{e}^{-\frac{{T}_{1}}{T+{T}_{0}}}\,$$

In this model, the conductance is separated into two terms, the first term scales linearly with temperature whilst the second term takes into account the fluctuation assisted tunnelling^49^. Here *σ*_1_ and *σ*_2_ are constants and *T*_1_ represents the activation energy required to tunnel through the barriers and *T*_0_ is the temperature at which the crossover from the saturating to activated transport occurs. This model has been successfully utilized in a range of disordered carbon networks. It should also be noted that there exists some reports^50^ on a combined FIT/variable range hopping (VRH) model which introduces a VRH term instead of the linear term in equation 2, as presented in the supplementary information figure S3 this does not fit as well to our data set. It should also be noted that some studies have linked the saturation in the resistance at low temperature to tunnelling between outer and secondary shells of the MWNT^15^. However, due to the divergence of the susceptibility and non-linearity of *I*-*V* characteristics which both occur at the low temperatures it is believed in this system the low temperature behaviour is due to electron and spin correlations. This led to probing the saturating resistance considering the numerical renormalized group (NRG) calculation as presented in Fig. 5b which shows the normalized resistance (with respect to the saturation value) as a function of temperature. A clear saturation is observed below approximately 4 K. The solid red line is a fit to the numerical renormalization group equation where *c* = 6.088 and ${T}_{K}$ is the Kondo temperature.

3 $$\frac{R(T)}{R(0)}=(1-c{(\frac{T}{{T}_{K}})}^{2})$$

From the fitting, *T*_K_ = 91 K is extracted although surprisingly high this value is very similar to what has recently been observed in disordered graphene using the same fitting^51^. Additionally, it is observed that the equation fits the data set best in the region below 14 K, signifying the crossover from the interrupted metallic transport (FIT fitting) at higher temperatures. The *T* ^2^ dependence of the resistance is a feature of magnetic impurities in a Fermi system, this finding is contrary to theoretical studies where a local non-fermi behaviour was expected for magnetic impurities linked to MWNTs which are expected to show a *T*^1/2^ dependence.

**Figure 6:**
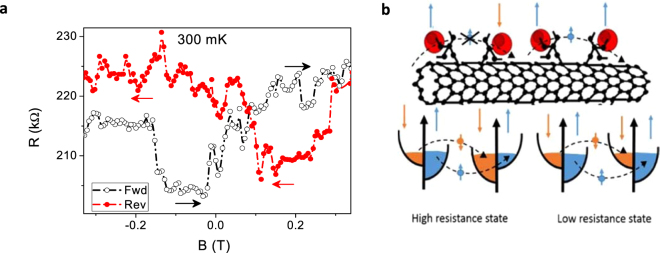
Spin switching behavior. (**a**) Magneto-resistance switching for the Gd-functionalized MWNT device showing symmetric switching within ±200 mT with respect to forward and reverse field sweeps similar to non-local spin valve behavior. There is a switching effect, roughly 8%, when current is 1 µA. (**b**) A schematic representing how neighboring Gd-DTPA molecular complexes can act as spin valve barriers effectively preventing or promoting transport of conduction electrons depending on local spin densities coupled to the Gd-DTPA complex analogous to a molecular spin valve.

To further probe the magnetic properties of the Gd-Fctn-MWNTs the dependence of the resistance on the magnetic field is investigated as shown in Fig. 6a. At low temperatures, below the resistance saturation, the magnetoresistance shows repeatable pronounced switching behaviour symmetric about the zero-field axis reminiscent of spin valve effects observed in other types of devices^29–31^. The spin switching effect, a sharp increase in resistance at certain fields, is clearly observed at 300 mK within ± 0.25 T which was determined to be the field at which saturation of the magnetic moment occurs as observed in the magnetic hysteresis (Fig. 4a). This device configuration is unlike the conventional CNT spin valve devices with ferromagnetic leads functioning as the spin polarizers. It consists of a non-magnetic CNT grafted with magnetic Gd-DTPA similar to the work on molecular magnets presented in ref.^29^. It is well known that in MWNT the conduction electrons are found in the outer shells (unlike in SWNTs), hence the close proximity of the conduction electrons and the magnetic entities results in their enhanced spin interaction. The neighbouring Gd-DTPA complexes can act as spin related barriers effectively suppressing or mediating transport of conduction electrons depending on the local spin densities due to the Gd-DTPA consequently forming a molecular spin valve. MR values of up to 8% are observed in the devices fabricated from the Gd-Fctn-MWNT which is interpreted as an effect of the collective switching of the Gd^3+^ magnetic domains on the aligned CNTs. In order to explain such features, we believe the resistance depends on the relative alignment (parallel and anti-parallel states) of the spin on the Gd^3+^ ions. As shown in Fig. 6b, the anti-aligned Gd ions form a higher resistive state that prevents the conduction of electrons between Gd-DTPA sites, by applying the magnetic field the spins can be switched to an aligned state, leading to a lower resistance where electron can more easily travel between Gd-DTPA sites. Similar results have been reported for CNT devices fabricated with arrays of CNTs with multiple nonlocal ferromagnetic contacts^26^, there however it was shown that the orientation of the different ferromagnetic contacts can change the switching fields and magnetoresistance difference quite drastically. We believe that this is the first report showing spin-valve like effect using a mesoscopic bundle of CNTs without ferro-magnetic contacts and is a clear indication that the functionalized CNTs can be useful for spin filtration/polarization devices, a pronounced feature of this strongly correlated system is the Kondo effect.

## Conclusion

We have successfully demonstrated that covalently functionalizing Gd-DTPA to MWNT yields a system with stable interaction between the host material and magnetic nanoclusters. We have characterized the supramolecular complex through a combination of HRTEM, Raman spectroscopy, superconducting quantum interference device (SQUID) magnetometry and transport measurements. We have observed an enhanced effective moment and non-superparamagnetic behaviour indicating strong spin interactions. The low field magnetoresistance shows clear switching spin valve behaviour which has not yet been reported for gadolinium modified CNT bundles. The electronic transport of the nanotubes is controlled by the magnetic states of the aggregated complex grafted onto the surface of the MWNTs. The covalent interactions allow for the effective mediation of the spin states from the magnetic complex to the CNT providing an alternative pathway for the relaxation of the Gd cluster magnetization. Currently 1-dimesntional semiconductors are at the forefront of many interesting scientific developments, most notably quantum computing, this works highlights the possibility of tailoring carbon nanotube quantum transport in ways that may find application in this emergent field.

## Methods

### Sample preparation

MWNTs were modified using a chemical functionalization route with a gadolinium complex, Diethylene triamine pentaacetic acid gadolinium (III) (DTPA). Commercial grade MWNTs from Sigma Aldrich were used. A suspension of 452 mg of DTPA and 46 mg of dry CNTs in Trifluoroacetic acid (TFA) (8 ml) is sonicated at 30 °C for 2 min to ensure even dispersion. The suspension is further stirred at room temperature for 20 h. After evaporation under reduced pressure, the residue is washed with diethylether, dichloromethane and methanol. The solid residue is then dried under reduced pressure. Chelation of Gd^3+^ is achieved by dispersing 10.53 mg of DTPA/CNTs in 12 ml of a gadolinium perchlorate 40% aqueous solution. The mixture was sonicated for 30 min and stirred at room temperature for 24 h. The suspension was centrifuged and the aqueous supernatant checked for free gadolinium ions by colorimetric detection with xylenol orange. The reaction product was dried under vacuum to obtain Gd-Fctn-MWNTs. All reactions were performed under inert conditions.

### Experimental Methods

Structural characterisation is done using HRTEM. The presence of Gd^3+^ in the nanocomposite is confirmed by EDS. The formation of the complex is investigated by Fourier transform Infrared spectroscopy (FTIR). Magnetic force microscopy (MFM) is used to investigate the existence of magnetic domains on the surface. Electronic transport studies are done on devices fabricated from the Gd-Fctn-MWNT composite. As shown in Fig. 1, HRTEM was used to investigate the morphology of the Gd-Fctn-MWNT. Quantification of the Gd concentration in the nanocomposites was done by a microwave-assisted HNO_3_/H_2_SO_4_ digestion (Ultra Wave Millestone) and analysis by ICP AES (ICAP 6500 Thermofischer Scientific). Raman spectroscopy was performed with an excitation wavelength of 514 nm. *M*(*H*) and *χ* = *M*(*T*)*/H*, (*H* = 100 Oe) studies of the composite were carried out at room temperature using an ultra-sensitive MPMS-SQUID magnetometer from Quantum Design, San Diego.

Dielectrophoresis (DEP) was used to fabricate Gd-functionalized MWNT network devices. The Gd-Fctn-MWNT were dispersed in isopropylalcohol and then sonicated for 6 hours. The resulting solution was drop cast on a prefabricated 6-gold-electrode system with a separation distance of approximately 5 and 1.5 μm between the furthest and closest electrodes respectively. The MWNTs are aligned by DEP using an alternating current of 1 MHz and ±5 *V*pp voltage. The outermost electrodes were used to contact a four-terminal device configuration using a wire bonder. Electronic transport characterization was done in the Cryogenic high field measurement system on different devices. Current (*I*)-voltage (*V*) measurements were done at room temperature and 300 mK. The Resistance vs Temperature measurements were carried out from 300 mK to 300 K using a Keithly 2400 to supply a current of 1 μA and a Keithly 2182 nanovoltmeter to measure the voltage across the sample. The magnetoresistance (MR) was measured from −0.5 to 0.5 T at excitation currents of at 300 mK.

## Electronic supplementary material

Supplementary Information
