# Supplementary material for: Kondo effect and enhanced magnetic properties in gadolinium functionalized carbon nanotube supramolecular complex
Source: Sci Rep. 2018 May 23;8:8057. doi: 10.1038/s41598-018-26428-y (PMC5966395; doi:10.1038/s41598-018-26428-y)
Supplement: Supplementary file 1 — Supplementary Information [file 41598_2018_26428_MOESM1_ESM.docx]

**Supplementary information**

**Kondo effect and enhanced magnetic properties in gadolinium functionalized carbon nanotube supramolecular complex**

**S. Ncube^a^, C. Coleman^a^, A. Strydom^b,c^, E. Flahaut^d^, A. de Sousa^e^, S. Bhattacharyya^a*^**

**^a^** Nano-Scale Transport Physics Laboratory, School of Physics, and DST/NRF Centre of Excellence in Strong materials, University of the Witwatersrand, Johannesburg, South Africa.

**^b^** Highly Correlated Matter Research Group, Department of Physics, University of Johannesburg, Auckland Park 2006, South Africa

**^c^** Max Planck Institute for Chemical Physics of Solids, Nothnitzer str. 40, D-01187 Dresden, Germany.

**^d^** CIRIMAT, Université de Toulouse, CNRS, INPT, UPS, UMR CNRS-UPS-INP N°5085, Université Toulouse Paul Sabatier, Bât. CIRIMAT, 118, route de Narbonne, 31062 Toulouse cedex 9, France.

**^e^** School of Chemistry, University of the Witwatersrand, Johannesburg, South Africa.

**Energy Dispersion Spectroscopy (EDS):**


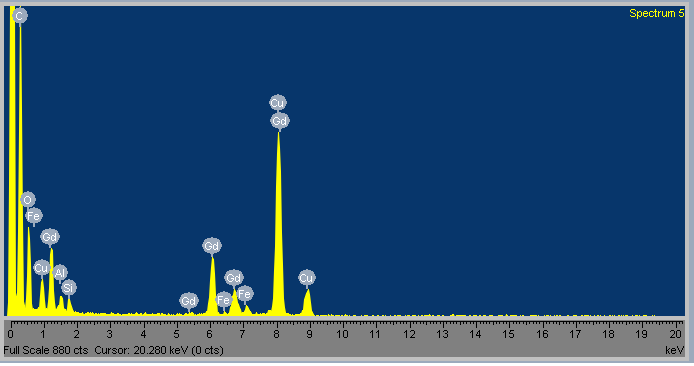

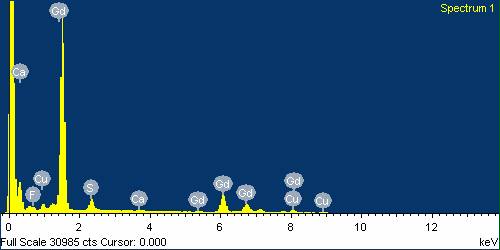


Figure S1. Energy dispersive spectra confirming the presence of Gd in the composite, top panel is EDS spectrum obtain through TEM, bottom panel is the EDS spectrum obtasined through SEM.

The EDS spectrum of the Gd-DTPA-MWNT complex was acquired with both TEM (top panel) and SEM (bottom panel). In both cases we observe the characteristic peaks for the Gd^3+^ ion. The peaks for the Copper are from the grid and chamber whereas the Iron peaks (Fe) are possibly from catalyst. However the digestive elemental analysis did not detect any appreciable amount of Iron we therefore attribute these peaks to contamination of the chamber.

**Susceptibility and inverse susceptibility GdDTPA**:

By comparing the results of SQUID magnetometry for the various samples we are able to establish the origin and type of magnetic interaction. Figure S2a, the Gd-DTPA control clearly showed paramagnetic susceptibility. The effective moment calculated from the inverse susceptibility of the Gd-DTPA was found to be 8.7 *µ*_B_ which is roughly 10 % larger than that of the bare Gd^3+^ ion theoretical value^s1^. But smaller than the typical experimental values. Modification of the Gd effective moment, both enhancement and reduction, has been observed in Gd-carbon systems and is generally attributed to correlation effects^s5^ between Gd ion and carbon lattice or molecule. The Increase in moment here indicates a ferromagnetic coupling between Gd and DTPA molecule^s2,s3,s4,s5^. As can be seen in figure S2b, the pristine MWNTs show a very small paramagnetic response this has been observed before and is attributed to ferromagnetic particles remaining from synthesis. The effect of these particles are however small when compared to the magnetic response of the Gd functionalized and filled tubes. In the study the molar susceptibility was used along with the Curie-Weiss law to determine the properties such as the Curie constant and Weiss temperature presented in the paper. This was done to allow for calculation of the effective moment which requires knowledge of molar concentration of the magnetic species.

**Figure S2.** The plot of temperature dependence on susceptibility and inverse susceptibility for **a)** Gd-DTPA molecular complex and **b)** pristine MWNTs.

The Gd-DTPA (Figure S2 a) which is paramagnetic differs significantly from the functionalized Gd-DTP-MWNT complex as presented in the paper, this is a strong indication that the resultant interaction observed is due to MWNT-Gd correlations and not dominated exclusively by the Gd-DTPA. This does present some difficulty in determining the effective moment of the respective samples as the susceptibility is presented in terms of number of moles of only the Gd found in each sample. The concentration was determined from the elemental analysis. This means, as stated in the manuscript, that the effective moment calculated in this way will lead to an over estimation as it does not take into account the carbon nanotube contribution.

**Supplementary Table S1: FTIR vibrational modes for the Gd-Fctn-MWNTs**

| **Peak (cm^-1^)** | **Designation** |
| --- | --- |
| 579 | Gd-O vibrations |
| 631 | Gd-O vibrations |
| 671 | Gd-O vibrations |
| 677 | Gd-N vibrations |
| 750 | Gd-N vibrations |
| 773 | Gd-N vibrations |
| 910 | CH_2_ deformation |
| 1026 | CH_2_ bending vibration |
| 1184 | –C-O stretching vibration |
| 1227 | –C-O stretching vibration |
| 1364 | COO^-^  stretching vibration |
| 1387 | COO^-^  stretching vibration |
| 1636 | –C=O stretching vibration |
| 1661 | –C=O stretching vibration |
| 1734 | –C=O acyl stretching vibration on surface. |
| 1998 | CNT |
| 2093 | CNT |
| 2120 | CNT |
| 2334 | CNT |
| 2344 | CNT |
| 2355 | CNT |
| 2668 | CNT |
| 3009 | C–H stretching vibration |
| 3017 | C–H stretching vibration |
| 3271 | O–H stretching vibration |
| 3402 | O–H stretching vibration |
| 3549 | O–H stretching vibration |

The table is a complete compilation of the FTIR active vibrational modes observed in the functionalized material after the Gd-DTPA has been attached to the MWNTs**.**

**Failure to fit transport data to Variable Range Hopping:**

There are many reports (as referenced in the main paper) reporting on variable range hopping being the expected transport mechanism in carbon nanotube films. However our films do not fit the VRH mechanism for 1, 2 or 3-dimentions as shown in figures S3 a-c. This deviation from expected behaviour is most likely due to the functionalization and in the main text we have shown a fit at higher temperatures to the thermal assisted tunnelling mechanism and a fit to the numerical renormalized group theory at low temperatures and the saturation point. This highlights the electron correlation effects we observe in the material.

b)

a)

c)

**Figure S3.** Conductance vs. temperature data with variable range hopping model (red line). The data is clearly not a good match to the VRH model for **(a)** 1-dimnensional **(b)** 2-dimensional or **(c)** 3-dimensional transport**.**

**References:**

s1 Li J., Wang T., Feng Y., Zhang Y., Zhen M., Shu C., Jiang L., Wang Y., and Wang C., A water-soluble gadolinium metallofullerenol: facile preparation, magnetic properties and magnetic resonance imaging application., Dalton Trans., **45**, 8696 (2016).

s2 Zhang J., Ye Y., Chen Y., Pregot C., Li T., Balasubramaniam S., Hobart D. B., Zhang Y., Wi S., Davis, Madsen L. M., Morris J. R., LaConte S. M., Yee G. T., and Dorn H. C., Gd3N@C84(OH)x: A New Egg-Shaped Metallofullerene Magnetic Resonance Imaging Contrast Agent, J. Am. Chem. Soc., **136**, 2630−2636 (2014).

s3 Kitaura R., Okimoto H., Shinohara H., Nakamura T., and Osawa H., Magnetism of the endohedral metallofullerenes M@C82 „M=Gd,Dy… and the corresponding nanoscale peapods: Synchrotron soft x-ray magnetic circular dichroism and density-functional theory calculations,.Phys. Rev. B **76**, 172409 (2007).

s4 Huang H. J. and Yang S. H., and Zhang X. X., Magnetic Behavior of Pure Endohedral Metallofullerene Ho@C82:  A Comparison with Gd@C82, J. Phys. Chem. B **103**, 5928-5932 (1999).

s5 Sabirianov R. F., Mei W. N., Lu J., Gao Y., Zeng X. C., Bolskar R. D., Jeppson P., Wu N., Caruso A. N., and Dowben P. A., Correlation effects and electronic structure of Gd@C60, J. Phys.: Cond. Matter., **19**, 082201 (2007).
